# Preferences of healthcare providers for capitation payment in Kenya: a discrete choice experiment

**DOI:** 10.1093/heapol/czaa016

**Published:** 2020-06-15

**Authors:** Melvin Obadha, Jane Chuma, Jacob Kazungu, Gilbert Abotisem Abiiro, Matthew J Beck, Edwine Barasa

**Affiliations:** c1 Health Economics Research Unit, KEMRI|Wellcome Trust Research Programme, P.O. Box 43640 – 00100, Nairobi, Kenya; c2 The World Bank, Kenya Country Office, P.O. Box 30577-00100, Nairobi, Kenya; c3 Department of Planning, Faculty of Planning and Land Management, University for Development Studies, Wa, Ghana; c4 Institute of Transport and Logistics Studies, Business School, The University of Sydney, Darlington, New South Wales 2006, Australia; c5 Centre for Tropical Medicine and Global Health, Nuffield Department of Medicine, University of Oxford, Peter Medawar Building for Pathogen Research, South Parks Road, Oxford OX1 3SY, UK

**Keywords:** Capitation, discrete choice experiment, provider payment mechanism, strategic purchasing

## Abstract

Provider payment mechanisms (PPMs) are important to the universal health coverage (UHC) agenda as they can influence healthcare provider behaviour and create incentives for health service delivery, quality and efficiency. Therefore, when designing PPMs, it is important to consider providers’ preferences for PPM characteristics. We set out to uncover senior health facility managers’ preferences for the attributes of a capitation payment mechanism in Kenya. We use a discrete choice experiment and focus on four capitation attributes, namely, payment schedule, timeliness of payments, capitation rate per individual per year and services to be paid by the capitation rate. Using a Bayesian efficient experimental design, choice data were collected from 233 senior health facility managers across 98 health facilities in seven Kenyan counties. Panel mixed multinomial logit and latent class models were used in the analysis. We found that capitation arrangements with frequent payment schedules, timelier disbursements, higher payment rates per individual per year and those that paid for a limited set of health services were preferred. The capitation rate per individual per year was the most important attribute. Respondents were willing to accept an increase in the capitation rate to compensate for bundling a broader set of health services under the capitation payment. In addition, we found preference heterogeneity across respondents and latent classes. In conclusion, these attributes can be used as potential targets for interventions aimed at configuring capitation to achieve UHC.

**Key Messages**

- Capitation arrangements with frequent payment schedules, timelier disbursements, higher payment rates per individual per year and paid for a limited set of services were preferred.
- The capitation rate per individual per year was the most important attribute according to senior health facility managers.
- Senior health facility managers were willing to accept an increase in the capitation rate to compensate for payment delays by more than three months as compared with those paid on time and to requite for bundling a broad set of health services compared with a limited one.
- There may be need to review capitation rates, improve timeliness of disbursements, increase frequency of payments and review the range of health services bundled under the capitation payments to create positive incentives for healthcare providers to deliver needed services and improve quality and efficiency.

## Introduction

Universal health coverage (UHC) has been reemphasized by Sustainable Development Goal 3.8 (United Nations, 2018). UHC calls for access to preventive, promotive, curative and rehabilitative services relative to need, quality and financial risk protection (World Health Organization, 2010). To achieve UHC, countries are reforming their health financing strategies; however, these reforms have focused on revenue collection and pooling functions of health financing (Mathauer *et al.*, 2019). One area that deserves attention in the reforms is purchasing; the transfer of pooled funds to healthcare providers to deliver health services to the population (Kutzin, 2001; Figueras *et al.*, 2005). Purchasing involves three key decisions: first, which health services are to be purchased; second, from which healthcare providers; and third, how to contract and pay the providers (PPMs) (Kutzin, 2001; Figueras *et al.*, 2005). Purchasing can be passive or active (strategic). In passive purchasing, information is not continuously used to allocate pooled funds, i.e. reimbursing bills without confirming or allocating budgets based on retrospective allotments (World Health Organization, 2000; Figueras *et al.*, 2005). Conversely, strategic purchasing involves structuring purchasing arrangements to promote health system objectives (such as efficiency, equity and quality of care) World Health Organization, 2000; Figueras *et al.*, 2005).

There is growing recognition of the importance of strategic purchasing in the achievement of UHC (Preker *et al.*, 2007; World Health Organization, 2010). Specifically, there is significant interest in the role of PPMs in influencing healthcare providers’ behaviour via incentives designed to encourage the provision of quality health services efficiently and equitably (Preker *et al.*, 2007; World Health Organization, 2010). One common PPM is capitation, which is a fixed payment to a healthcare provider to deliver a set of services to an enrolee for a specified time period (Langenbrunner *et al.*, 2009). Capitation is important as it creates incentives for healthcare providers to control costs and improve efficiency but may result in the under-provision of services (Brocklehurst *et al.*, 2013; Cashin, 2015).

In Kenya, capitation is mainly used by the National Hospital Insurance Fund (NHIF) to pay for outpatient health services (Barasa *et al.*, 2018). The NHIF, the Kenyan social health insurance scheme, contracts private (for-profit and not-for-profit), public, faith-based and non-governmental organization (NGO) healthcare providers in a public contract model (Munge *et al.*, 2018). The NHIF is compulsory for people in formal employment but voluntary for the rest. NHIF enrolees select and register at a healthcare provider where they receive outpatient services. The provider then receives capitation payments for that enrolee on a quarterly basis to provide a predetermined set of outpatient services. The outpatient services usually include consultation, basic diagnostic tests such as laboratory services and X-rays, drugs that are specified in the Kenya Essential Drug List, vaccinations, same-day procedures, health education, counselling and physiotherapy (National Hospital Insurance Fund, 2019). Healthcare providers are expected to provide a standard and similar range of services. If certain services are unavailable in the health facility (e.g. certain laboratory services), the facility is required to outsource this service at zero cost to the patient by getting into a service-level agreement with a facility that provides the service. The NHIF pays Kenya Shillings (KES) 1200 (US $12) per year for an enrolee under the general scheme. Under the civil servants’ scheme, NHIF pays public healthcare providers KES 1500 (US $15) per enrolee per year while private providers receive KES 2850 (US $28.50) per year (Barasa *et al.*, 2018). Studies on the experiences of healthcare providers with capitation in Kenya revealed that the payment scheme was perceived as an important contributor to the providers’ overall revenues (Sieverding *et al.*, 2018; Obadha *et al.*, 2019c); however, delayed payments and inadequate capitation rates were reported as negative experiences (Sieverding *et al.*, 2018; Obadha *et al.*, 2019c).

Kenya has made a political commitment to achieve UHC by 2022 and is reforming its health financing strategy. However, when designing capitation schemes, preferences of healthcare providers for the characteristics (attributes) of PPMs are rarely taken into consideration (Munge *et al.*, 2018; Obadha *et al.*, 2019c). Furthermore, healthcare providers are hardly involved in the design process. Therefore, to design efficient PPMs that will create the right incentives for healthcare providers, it is important to identify providers’ preferences for the attributes of capitation, especially those they consider important. These attributes can subsequently be used as target points for interventions meant to configure the capitation payment system.

Stated preference elicitation methods such as discrete choice experiments (DCEs) can be used for this purpose, an econometric technique used to elicit preferences (Hensher *et al.*, 2015). Respondents in a DCE are asked to select an alternative they prefer among a set of two or more hypothetical alternatives (Hensher *et al.*, 2015). Each alternative is defined by a set of two or more attributes and their corresponding levels. DCEs are used for valuation of attributes and estimation of marginal rates of substitution (MRS) of one attribute over another (also known as trade-offs).

DCEs have theoretical underpinnings from Lancaster’s theory of consumer demand and random utility theory (RUT). Lancaster’s theory states that goods and services are defined by their attributes (Lancaster, 1966). Therefore, individuals derive utility not from the goods or services but from their underlying attributes. Utility theory states that individuals are utility maximizers and will select the good or service that gives them the highest utility (McFadden, 1973). From the choices the respondents make in a DCE, researchers can quantify the relative importance the respondents place on the attributes of the goods or services being considered and compute MRS estimates. When MRS values are expressed in monetary terms, they are known as marginal willingness to pay (WTP) or willingness to accept (WTA) estimates (Hensher *et al.*, 2015).

While several studies in low- and middle-income (LMIC) settings have explored healthcare providers’ views of—and experiences with—capitation payment schemes (Agyei-Baffour *et al.*, 2013; Andoh-Adjei *et al.*, 2018; Sieverding *et al.*, 2018; Suchman, 2018; Wangai *et al.*, 2019), very few have sought to quantify provider preferences for the payment mechanism. A notable exception is Robyn *et al.* (2012) who conducted a DCE among health workers in Burkina Faso to elicit their preferences for the attributes of capitation used in a community-based health insurance (CBHI) scheme. The researchers found that schemes with higher capitation payment levels, frequent payments and reimbursed service fees were preferred. Though the study provided useful information on the preferences of health workers for capitation attributes, the researchers only examined payment mechanisms used specifically for a CBHI scheme and based their study on a single district in Burkina Faso. Furthermore, different capitation attributes need to be studied in other contexts to generate global evidence that needs to be considered when designing capitation payment schemes.

To bridge this gap in literature and generate evidence that is policy relevant in the current context of health financing reforms in Kenya, we conducted a DCE to elicit the preferences of senior health facility managers for the attributes of capitation payment mechanism in Kenya. Healthcare providers (also known as health facilities) are organizations providing health services and consist of individuals employed to work within them. The individuals who lead these organizations are known as senior health facility managers. A DCE was the ideal technique as it would not only enable us to elicit senior health facility managers’ preferences but also quantify the relative importance they placed on the capitation attributes and compute marginal WTA estimates. Furthermore, a DCE would enable us to explore heterogeneity in preferences.

## Methods

### Study setting and design

Kenya is a lower middle-income country with a central (national) government and 47 semi-autonomous units referred to as counties (Government of Kenya, 2010). The health system is organized in four tiers, namely, community, primary care, county (secondary care) and national (tertiary care) levels (Ministry of Health Kenya, 2014). First, the community level creates demand for healthcare services and identifies individuals to be referred to primary care level. Second, primary care level consists of clinics, dispensaries, health centres and maternity homes that provide basic outpatient and short-stay inpatient services like deliveries. Third, secondary care-level consists of public, large faith-based, and large private facilities that provide inpatient, outpatient, and specialized services and some public hospitals that conduct teaching and research. Finally, tertiary care level comprises national public referral hospitals that offer highly specialized services and conduct research and training for the whole country and also comprises very large private and faith-based facilities (Ministry of Health Kenya, 2014).

In the public sector, county governments oversee the running of dispensaries and health centres that provide outpatient primary care services. Furthermore, the county governments run county hospitals that provide secondary outpatient and inpatient care. The national government has policy and regulatory roles, as well as provides tertiary-level care in national referral hospitals. Conversely, private entities/individuals own private (for-profit and not-for-profit) health facilities while religious organizations or NGOs run faith-based/NGO facilities. Overall, there are more than 11 000 health facilities in Kenya with 5300 of them being NHIF accredited (Ministry of Health Kenya, 2019; National Hospital Insurance Fund, 2019).

The Kenyan health system has multiple purchasers that include CBHI organizations, county governments, the national government, NHIF and private health insurance (PHI) companies among others (Munge *et al.*, 2018). In the public sector, secondary care-level health facilities receive line-item budgets, medical equipment and supplies (e.g. drugs) and staff salaries from county governments (Mbau *et al.*, 2018). They are also contracted by NHIF and paid using capitation for outpatient services, case-based payments for specialized and maternal services, per diem for inpatient services and fee-for-service (FFS) for both outpatient and inpatient services (Munge *et al.*, 2018). In addition, they receive out-of-pocket payments from patients. Public tertiary-level health facilities receive global budget allocations from the national government and out-of-pocket payments from patients and are also contracted by NHIF. Contrarily, public primary care-level health facilities receive line-item budget allocations, medical equipment and supplies and staff salaries from county governments and case-based payments from the NHIF for maternity services. They are neither contracted by NHIF nor do they charge user fees and solely depend on the county governments for their finances. Finally, private and faith-based/NGO primary, secondary and tertiary health facilities have multiple sources of revenues such as CBHI, NHIF, PHI and out-of-pocket payments from patients. They do not receive funds or supplies from the national or county governments and, therefore, have to use their revenues to pay salaries, purchase drugs and medical equipment.

We conducted a cross-sectional DCE survey in seven counties, namely, Bomet, Kakamega, Kilifi, Makueni, Meru, Migori and Siaya (Table 1). These counties were randomly selected from the 47 counties in the country.

**Table 1:** County statistics

County	Projected population (2014) (Kenya National Bureau of Statistics, 2015)	Total number of health facilities (2019) (Ministry of Health Kenya, 2019)	Number of faith-based and NGO health facilities (Ministry of Health Kenya, 2019)	Number of private health facilities (Ministry of Health Kenya, 2019)	Number of public health facilities (Ministry of Health Kenya, 2019)	County expenditure on health as a percentage of total county expenditure for the first half of the 2018/19 financial year (Office of the Controller of Budget Kenya, 2019) (%)
Bomet	861 396	174	7	28	139	22.07
Kakamega	1 812 330	314	29	115	170	10.04
Kilifi	1 307 185	334	29	164	141	34.06
Makueni	939 879	345	29	76	240	41.74
Meru	1 441 361	540	67	299	174	39.99
Migori	1 025 422	268	36	86	146	32.37
Siaya	941 724	234	27	63	144	32.20
Total	8 329 297	2209	224	831	1154	

In conducting the DCE, we followed the International Society for Pharmacoeconomics and Outcomes Research Conjoint Analysis Task Force checklist for good research practices (Bridges *et al.*, 2011).

### Attributes and levels

To derive the attributes and levels, we followed Helter and Boehler (2016) four-stage process that consisted of raw data collection, data reduction, removing inappropriate attributes and wording of attributes. The first two stages, raw data collection and reduction, were accomplished by conducting a literature review and qualitative study. The literature review derived eight broad attributes of PPMs that influenced healthcare provider behaviour from 16 peer-reviewed journal articles (Kazungu *et al.*, 2018). The qualitative study involved semi-structured interviews with 29 senior health facility managers and generated ten capitation attributes and levels (Obadha *et al.*, 2019c).

The third stage incorporated a panel of eight experts who reduced the long list of capitation attributes and levels to seven using criteria such as appropriateness, relevance, the capability of being traded, salience, inter-attribute correlation and plausibility (Abiiro *et al.*, 2014; Helter and Boehler, 2016). In the fourth stage, three researchers deliberated and agreed on an interim list of five attributes and levels. These were tested in a pilot study with 31 senior health facility managers (Obadha *et al.*, 2019b). Then, six researchers reviewed the pilot study results and agreed on a final list of four attributes (Table 2), namely, payment schedule, timeliness of payments, capitation rate per individual per year and services to be paid by the capitation rate. We comprehensively described our attribute development and level selection process in an earlier publication (Obadha *et al.*, 2019a).

**Table 2:** Capitation attributes and levels

Attributes	Levels	Definition	Attribute type
Payment schedule	1 month (every month)	Frequency of capitation disbursements	Continuous
	3 months (every quarter)		
	6 months (twice a year)		
	12 months (once a year)		
Timeliness of payments	Delayed by more than 3 months	Timeliness of capitation payments	Discrete
	Delayed by less than 3 months		
	Timely		
Capitation rate per individual per year (shillings)^a^	800	The capitation amount the health facility will receive in advance for an enrolee per year	Continuous
	1600		
	2400		
	3200		
Services to be paid by the capitation rate	Consultation only	The outpatient service package the health facility will provide to an enrolee that is paid for using capitation	Discrete
	Consultation and laboratory tests		
	Consultation and drugs		
	Consultation, laboratory tests, drugs, and imaging (e.g. X-rays)		

^a^US $ 1 = KES 100.

### Construction of choice sets and experimental design

In the construction of choice sets, we used full profiles that contained all four attributes. A choice set (Table 3) consisted of three alternatives: two unlabelled alternatives (Capitations A and B) and an opt-out labelled ‘neither’. Respondents would be prompted to rank their preferences from best (1) to worst (3). The opt-out (no-choice alternative) was included to reflect the real market scenario as senior health facility managers could opt-out of the capitation scheme (Determann *et al.*, 2019).

**Table 3:** Sample choice set

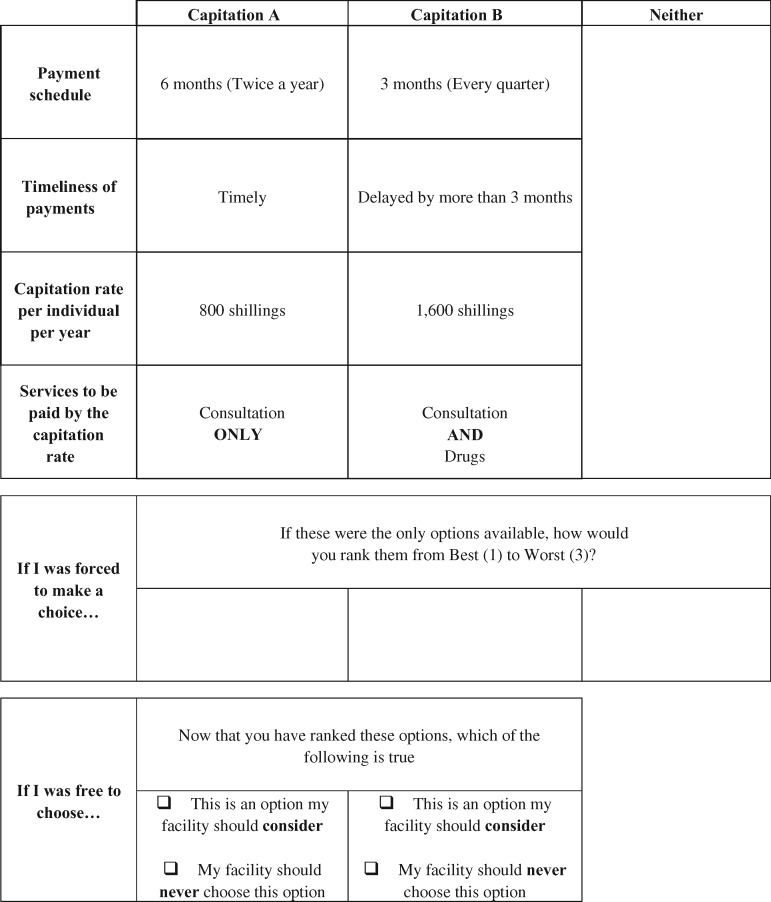

Twelve choice sets were deemed optimal for the main survey as respondents easily handled eight during the pilot study (Obadha *et al.*, 2019a). Too many choice sets increase complexity and place a cognitive burden on the respondents, thereby negatively affecting the response reliability (Bridges *et al.*, 2011). A fractional factorial experimental design, which takes a subset of the choice sets from the full factorial, was used as it reduces the number of choice sets the study respondents will face (Reed Johnson *et al.*, 2013). A Bayesian D-efficient design, a type of a fractional factorial design, was generated using the Ngene software version 1.2.0 (ChoiceMetrics, 2018). The Bayesian D-efficient design was selected over an orthogonal design as it captures more information, generates smaller standard errors and provides reliable parameter estimates using smaller sample sizes (Bliemer *et al.*, 2008; Rose and Bliemer, 2009). Furthermore, a Bayesian D-efficient design is less sensitive to misspecification of priors as compared with a D-efficient design (Bliemer and Collins, 2016).

Bayesian priors were obtained from a pilot study (Obadha *et al.*, 2019b). In the utility function, we specified a main effects model optimizing for a multinomial logit and evaluating for a panel mixed multinomial logit (MMNL) model. The categorical attributes, timeliness of payments and services to be paid by the capitation rate were estimated non-linearly. All Bayesian priors were assumed to be normally distributed, and three Gaussian draws per parameter were specified. Gaussian draws are recommended for Bayesian efficient designs as they provide more efficient estimates than Halton, modified Latin hypercube sampling or Sobol draws (Bliemer *et al.*, 2008). Each generated choice set was examined for the presence of a dominant alternative; none were found. Overall, the experimental design process resulted in the generation of 12 choice sets, with each respondent receiving all 12. As previously stated, our pilot study revealed that respondents were easily able to complete eight choice sets so the decision to move to 12 was considered practicable.

### Questionnaire design

The survey was a paper-based questionnaire and contained six sections. The first section collected information about the health facility while the second section captured socio-demographic characteristics of the respondent. The third section provided respondents with an introduction to the capitation method of payment. The fourth section explained the choice scenario and capitation attributes under consideration and provided two practice choice sets. The fifth section presented the respondents with 12 choice sets. Finally, the sixth section collected supplementary information. The questionnaire had been pretested in a pilot study and revisions made (Obadha *et al.*, 2019a).

### Sampling strategy and data collection

A stratified random sampling approach without replacement was adopted and targeted senior health facility managers. Seven counties (survey domains) were selected from a list of all 47 counties in Kenya using simple random sampling with equal probability of selection, i.e. equal probability simple random sampling. In each survey domain, two sampling strata were created (primary and secondary care-level health facilities). None of the selected survey domains had tertiary care-level health facilities. The sampling frame was obtained from the Kenya master health facility list, which is a register of all health facilities in the country (Ministry of Health Kenya, 2019). The number of health facilities sampled in each survey domain was determined using power allocation with power value *α* set at 0.5, i.e. square root allocation (Bankier, 1988). This approach ensured that small domains were over sampled and large ones under sampled with the power value *α* of 0.5 (Bankier, 1988).

In each stratum, an equal number of primary and secondary care-level health facilities (equal allocation) were selected using simple random sampling. A total of 99 health facilities were approached to participate through their institutional heads using emails, phone calls and face-to-face visits. In each health facility, a maximum of three senior management staff whose roles included financial decision-making were targeted, i.e. head of the facility, head of administration/operations and head of finance/accounts. We approached a total of 246 eligible individuals to participate.

Overall, 233 senior managers in 98 health facilities completed the DCE paper questionnaire at their workplace in the presence of the data collector after providing written informed consent. The response rate was 94.72%. Those who did not participate were either busy or on different kinds of leave. The data collectors took the respondents through the paper questionnaire including two practice choice tasks.

### Statistical analyses

RUT is the framework for the analysis of choice data. Utility *U_ijs_* of an alternative *j* in a choice set *s* can be additively broken down into a systematic and a random component (1) (McFadden, 1973; Hauber *et al.*, 2016):

(1) $$U_{\mathrm{ijs}}=\;V_{\mathrm{ijs}}+\;\varepsilon_{\mathrm{ijs}.}$$

The systematic component *V_ijs_* is explainable and consists of the attributes of the alternatives and the characteristics of individual *i* (summarized as *V_ijs_* = *β*ʹ*X_ijs_* where *X_ijs_* is a vector of attribute levels of alternative *j* and *β* is a vector of coefficients representing the preference weights). Conversely, the random part *ε_ijs_* is unexplainable and consists of the error terms, which are assumed to be independently and identically distributed (IID) across alternatives and respondents approximately following an extreme value distribution (Gumbel) with location parameter *η* and scale parameter *μ* [summarized as EV (*η*, *μ*), with *μ* > 0] (McFadden, 1973; Ben-Akiva and Lerman, 1985; Hensher *et al.*, 2015).

The assumption is that individual *i* will choose alternative *j* if and only if it maximizes their utility among all other alternatives in choice set *s*. Therefore, the probability *P_ijs_* that an individual *i* will choose alternative *j* over any other alternative *k* in choice set *s* can be represented as follows:

(2) $$P_{\mathrm{ijs}}=\mathrm{Pr}\left(U_{\mathrm{ijs}}>U_{\mathrm{iks}}\right)=\mathrm{Pr}\left(V_{\mathrm{ijs}}+\varepsilon_{\mathrm{ijs}}\;>\;V_{\mathrm{iks}}+\varepsilon_{\mathrm{iks}}\right)=\mathrm{Pr}\left(V_{\mathrm{ijs}}-V_{\mathrm{iks}}>\varepsilon_{\mathrm{iks}}-\varepsilon_{\mathrm{ijs}}\right)\forall k\neq j\in S.$$

If we assume that the error terms *ε*_ijs_ are IID, then we derive the following conditional logit model:

(3) $$P_{\mathrm{ijs}}=\frac{{\exp(\beta ,X}_{\mathrm{ijs}})}{\sum_{k\in S}\exp(\beta ,X_{\mathrm{iks}})}.$$

However, the conditional logit model is dependent on the independence of irrelevant alternatives (IIA) assumption holding (proportional substitution across alternatives) and does not account for heterogeneity in preferences across respondents (Hensher *et al.*, 2015; Hauber *et al.*, 2016; Lancsar *et al.*, 2017). Therefore, a panel MMNL model was preferred over the conditional logit model as it accounts for preference heterogeneity and relaxes IIA (McFadden and Train, 2000). Panel MMNL also accounts for within respondent correlation, i.e. the panel structure of our data as one respondent faces 12 choice sets.

Panel MMNL assumes that some or all the parameters *β* are randomly distributed following a specified probability distribution (McFadden and Train, 2000; Hensher *et al.*, 2015). Therefore, the probability *P_ijs_* that individual *i* will choose alternative *j* over any other alternative *k* in choice set *s* can be represented as follows:

(4) $$P_{\mathrm{ijs}}=\int_{\beta }P_{\mathrm{ijs}}\left(\beta ,X_{\mathrm{ijs}}\right)f\left(\beta |\Omega \right)d\beta ,$$

where f(*β|Ω*) is the multivariate probability density function of *β* given the distributional parameters *θ*, which can, e.g. be the means and standard deviations in the population (Hess *et al.*, 2005; Hensher *et al.*, 2015).

The utility function of our DCE was written as follows:

(5) $$U_{\mathrm{ijs}}=\beta_{0}{\mathrm{opt}-\mathrm{out}}_{\mathrm{ijs}}+\beta_{1}{\mathrm{payment}\;\mathrm{schedule}}_{\mathrm{ijs}}+\beta_{2}{\mathrm{delayedpayments}<3\mathrm{months}}_{\mathrm{ijs}}+\beta_{3}{\mathrm{delayedpayments}>3\mathrm{months}}_{\mathrm{ijs}}+\beta_{4}{\mathrm{capitationrate}}_{\mathrm{ijs}}+\beta_{5}{\mathrm{laboratory}}_{\mathrm{ijs}}+\beta_{6}{\mathrm{drugs}}_{\mathrm{ijs}}+\beta_{6}{\mathrm{imaging}}_{\mathrm{ijs}}+\varepsilon_{\mathrm{ijs}}.$$

The opt-out represents the no-choice alternative taking the value of one if neither was ranked first and zero otherwise. *β*_0_ was the alternative specific constant of the opt-out. Payment schedule was a continuous variable representing the frequency of capitation disbursements in months. Delayed payments <3 months and delayed payments >3 months were dummy variables for the timeliness of payment attribute with the reference level being timely payments. Capitation rate was the monetary attribute and specified as continuous in KES. Laboratory, drugs and imaging were dummy variables of the services to be paid by the capitation rate attribute with the reference level being consultation only. The parameter of the opt-out was fixed with all the rest assuming a random and normal distribution except for the monetary attribute (capitation rate per individual per year), which was restricted to a lognormal distribution. Lognormal distribution was used for the parameter of the capitation rate as a positive sign was expected for every respondent. A normal distribution is unbounded and may have resulted in both positive and negative coefficient estimates (Hess *et al.*, 2005). We used 1000 Halton draws for all panel MMNL models.

We tested for several interactions between the capitation attributes and respondents’ characteristics as identified in literature (Robyn *et al.*, 2012; Sieverding *et al.*, 2018; Obadha *et al.*, 2019c). These included capitation rate with gender, and with the ownership of the health facility the respondent worked in. Moreover, interactions were tested between services to be paid by the capitation rate attribute with the level of care the respondent worked in and with the ownership of the health facility the respondent worked in. Finally, we also tested for the interaction between the opt-out and ownership of the health facility the respondent worked in.

The estimates of the relative importance the respondents placed on the attributes were derived using the coefficients of the means from the panel MMNL model. We multiplied the absolute value of the coefficient of the mean of each attribute with the difference between the highest and lowest levels of the attribute to get the maximum effect (Maaya *et al.*, 2018). Then, the ratio between the maximum effect of each attribute and the total was computed to derive the relative importance scores. This method was preferred as it takes into account the fact that the coefficients of the attributes were estimated on different scales, i.e. two attributes were continuous and the other two were categorical.

In addition, we computed the marginal WTA estimates using a panel MMNL model in WTP space (Train and Weeks, 2005). Estimating marginal WTA estimates in WTP space results in realistic values as compared with estimating them in preference space (i.e. taking the ratio between the negative coefficient of the non-monetary attributes and the monetary one) (Train and Weeks, 2005; Hole and Kolstad, 2012). In our case, the monetary attribute (i.e. capitation rate per individual per year) was the amount of money senior health facility managers expected to receive for an enrolee who had registered at their health facility. This was assumed to be random and lognormally distributed. The parameter of the opt-out was fixed with all the rest assuming a random and normal distribution. The estimates were expressed in KES. We specified utility in WTP space as follows;

(6) $$U_{\mathrm{ijs}}=\beta_{4i}({\mathrm{capitationrate}}_{\mathrm{ijs}}+\beta_{0i}{\mathrm{opt}-\mathrm{out}}_{\mathrm{ijs}}+\beta_{1i}{\mathrm{paymentschedule}}_{\mathrm{ijs}}+\beta_{2i}{\mathrm{delayedpayments}<3\mathrm{months}}_{\mathrm{ijs}}+\beta_{3i}{\mathrm{delayedpayments}>3\mathrm{months}}_{\mathrm{ijs}}+\beta_{5i}{\mathrm{laboratory}}_{\mathrm{ijs}}+\beta_{6i}{\mathrm{drugs}}_{\mathrm{ijs}}+\beta_{6i}{\mathrm{imaging}}_{\mathrm{ijs}})+\varepsilon_{\mathrm{ijs}}.$$

To further explore preference heterogeneity and test the robustness of our results, we fitted a standard latent class logit model via the expectation–maximization algorithm with fixed parameters. A latent class model assumes that there are a finite number of segments (classes) *q* in the population (Hess, 2014; Hensher *et al.*, 2015). Preferences are homogenous within classes but heterogeneous across classes. Therefore, latent class model relaxes IIA across classes. However, we do not know which observations fall into which class and hence the term latent class. Therefore, we can model the probability of individual *i* selecting choice *j* in choice set *s* conditional on belonging to class *q* as follows:

(7) $$P_{is}\left(j|\beta_{1},\ldots ,\beta_{Q}\right)=\sum_{q=1}^{Q}\pi_{iq}P_{is}\left(j|\beta_{q}\right),$$

where Π_*iq*_ is the probability that individual *i* belongs to class *q* is given by

(8) $$\pi_{iq}=\frac{{\exp(Z_{i}^{\prime }\theta }_{q})}{\sum_{q=1}^{Q}\left(Z_{i}^{\prime },\theta_{q}\right)},$$

where *Z_i_* is the vector of characteristics of individual *i* that are used in class allocation and *θ_q_* is the vector of parameters to be estimated. Akaike information criterion, Bayesian information criterion and consistent Akaike information criteria were used to determine the optimal number of classes. All analyses were conducted on STATA 15.1 (StataCorp, 2017). Mixlogit command was used for the panel MMNL model, and lclogit was used for the latent class model (Hole, 2007; Pacifico and Yoo, 2013). Marginal WTA estimates in WTP space were computed using mixlogitwtp command (Hole, 2016).

## Results

### Descriptive statistics

Senior health facility managers had a median age of 35 years [inter-quartile range (IQR) 30–45]. Furthermore, more than two-thirds were male and 54.51% worked in public sector facilities (Table 4). Moreover, the median amount of professional work experience was 8 years (IQR 4–15) and 38.20% of the respondents were in-charge of their health facilities. Finally, 63.52% worked in health facilities that were receiving capitation payments.

**Table 4:** Characteristics of senior health facility managers

Characteristics	Proportion	*N*
Gender (%)		
Male	69.53	162
Female	30.47	71
		233
Age (years)		
Mean (standard deviation)	37.83 (10.07)	230
Median (inter-quartile range)	35 (30–45)	
Respondent’s profession (%)		
Medical doctor	12.02	28
Nurse	17.60	41
Clinical officer	18.88	44
Medical laboratory technologist/technician	1.72	4
Pharmacist/pharmaceutical technologist	1.72	4
Administration	21.03	49
Dentist	0.43	1
Accountant	19.74	46
Others	6.87	16
		233
Respondent’s job title at the health facility (%)		
Head of the facility (CEO/MD/in-charge)	38.20	89
Head of administration/operations	37.34	87
Head of finance/accounts	24.46	57
		233
Ownership of the health facility the respondent worked in (%)		
Private (for-profit and not-for-profit)	24.89	58
Public	54.51	127
Faith-based and NGOs	20.60	48
		233
Level of care the respondent worked in (%)		
Primary care level	42.92	100
Secondary care level	57.08	133
		233
Professional experience (years)		
Mean (standard deviation)	11.08 (9.50)	233
Median (inter-quartile range)	8 (4–15)	
Work experience at the current health facility (years)		
Mean (standard deviation)	3.86 (4.35)	232
Median (inter-quartile range)	3 (1–5)	
Whether the respondent had heard of capitation (%)		
No	14.59	34
Yes	85.41	199
		233
Whether the respondent worked in a health facility that received capitation (%)		
Never received	34.76	81
Used to receive	1.72	4
Currently receives	63.52	148
		233

### Preference and marginal WTA estimates

The coefficients of means of the attributes in the main effects panel MMNL model in preference space had the expected signs including the opt-out (Table 5). Capitation alternatives with infrequent payment schedules, delayed disbursements and paid for a service package that contained a broad range of health services were associated with a lower preference. Conversely, capitation alternatives that paid higher rates per individual were preferred. In addition, senior health facility managers preferred some form of capitation to nothing at all as manifested by the negative sign of the coefficient of the mean of the opt-out. Overall, there was inter-respondent heterogeneity in preferences across all attributes as suggested by the statistically significant standard deviation estimates.

**Table 5:** Main effects panel MMNL model preference weights and marginal WTA estimates

Capitation attributes	Preference estimates		Marginal WTA estimates in WTP space	
	Coefficient	S.E.	Coefficient	S.E.
Payment schedule *μ*	−0.1772^a^	0.0195	294.3163^a^	45.7672
Payment schedule *σ*	0.1962^a^	0.0218	347.4121^a^	49.8520
Timeliness of payment				
Timely	Ref. (0)		Ref. (0)	
Delayed by less than 3 months *μ*	−0.5915^a^	0.1222	955.4373^a^	212.7003
Delayed by less than 3 months *σ*	−0.8673^a^	0.1593	1241.2360^a^	227.2873
Delayed by more than 3 months *μ*	−1.4684^a^	0.1564	2151.5630^a^	277.1859
Delayed by more than 3 months *σ*	1.5365^a^	0.1681	2678.5530^a^	409.1235
Capitation payment rate per individual per year *μ*	0.0009^a^	0.0001	637.6078^a^	73.9337
Capitation payment rate per individual per year *σ*	0.0018^a^	0.0005	287.4414^a^	89.4012
Services to be paid by the capitation rate				
Consultation only	Ref. (0)		Ref. (0)	
Consultation and laboratory *μ*	−0.0917	0.1276	202.8751	215.9658
Consultation and laboratory *σ*	0.8484^a^	0.1741	1452.1250^a^	319.7487
Consultation and drugs *μ*	−0.1188	0.1496	197.1068	2392.9210
Consultation and drugs *σ*	1.0600^a^	0.1721	−1876.0030^a^	335.8870
Consultation, laboratory, drugs and imaging *μ*	−0.7157^a^	0.2384	961.1945^a^	385.1153
Consultation, laboratory, drugs and imaging *σ*	2.2820^a^	0.2623	3638.5650^a^	543.8956
Opt-out	−0.3456	0.2145	504.5644	460.7466
Model fit statistics				
Log-likelihood (final)	−2270.9846		−2368.2982	
Observations	8388		8388	
Number of decision-makers (*n*)	233		233	
Draws (Halton)	1000		1000	

a The 95% confidence interval does not include zero. μ is the mean while σ is the standard deviation. S.E. represents robust standard errors. The coefficients of capitation payment rate per individual per year were restricted to a lognormal distribution in preference and WTP space. All other coefficients were normally distributed except the opt-out that was fixed. Marginal WTA estimates are in KES. US $ 1 = KES 100.

The marginal WTA estimates in WTP space indicated that senior health facility managers needed a KES 294 (US $2.94) increase in the capitation rate per individual per year to accept an increase in the payment schedule by one month (Table 5). Furthermore, they required a KES 961 (US $9.61) increase in the capitation rate to compensate for bundling a broad set of health services (consultation, laboratory tests, drugs and imaging) as compared with a narrow set consisting of consultation only. Conversely, they were willing to accept a KES 2152 (US $21.52) increase in the capitation rate to compensate for payments delayed by more than 3 months as compared with timely disbursements. Finally, there was significant inter-respondent heterogeneity in the marginal WTA estimates across all attributes.

The relative importance scores revealed that capitation rate per individual per year was the most important attribute followed by payment schedule and timeliness of payments (Table 6). Services to be paid by the capitation rate was the least important attribute.

**Table 6:** Relative importance scores

Capitation attribute	Effect	Maximum effect	Relative importance
Payment schedule	0.1772	1.9492	0.3119
Timeliness of payments	1.4684	1.4684	0.2350
Capitation rate per individual per year	0.0009	2.1163	0.3386
Services to be paid by the capitation rate	0.7157	0.7157	0.1145
Total		6.2496	

### Interaction effects

The interaction term between capitation rate per individual per year and ownership of the health facility the respondent worked in suggested that if everything was held constant, then managers who worked in public health facilities had a significant preference for lower capitation rates per individual as compared with managers who worked at private facilities (Table 7). Furthermore, female managers manifested a lower preference for higher capitation rates than their male counterparts if everything was held constant as suggested by the interaction between capitation rate per individual per year and gender. However, the interaction was not statistically significant.

**Table 7:** Panel MMNL model preference estimates with interactions

Capitation attributes	Coefficient	S.E.
Payment schedule *μ*	−0.1596^a^	0.0180
Payment schedule *σ*	0.1671^a^	0.0175
Timeliness of payment		
Timely	Ref. (0)	
Delayed by less than 3 months *μ*	−0.5745^a^	0.1188
Delayed by less than 3 months *σ*	0.7930^a^	0.1630
Delayed by more than 3 months *μ*	−1.4845^a^	0.1592
Delayed by more than 3 months *σ*	1.2855^a^	0.1278
Capitation payment rate per individual per year *μ*	0.0008^a^	0.0001
Capitation payment rate per individual per year *σ*	0.0009^a^	0.0002
Capitation payment rate per individual per year × female *μ*	−0.0029	0.0183
Capitation payment rate per individual per year × female *σ*	1.6957	30.7269
Capitation payment rate per individual per year × respondent works in a public health facility *μ*	−0.0007^a^	0.0003
Capitation payment rate per individual per year × respondent works in a public health facility *σ*	0.0137	0.0217
Capitation payment rate per individual per year × respondent works in a faith-based/NGO health facility *μ*	0.0001	0.0001
Capitation payment rate per individual per year × respondent works in a faith-based/NGO health facility *σ*	0.0004	0.0003
Services to be paid by the capitation rate		
Consultation only	Ref. (0)	
Consultation and laboratory *μ*	0.3680	0.2728
Consultation and laboratory *σ*	0.6375^a^	0.1736
Consultation and laboratory × respondent works in a public health facility *μ*	0.4835	0.3177
Consultation and laboratory × respondent works in a public health facility *σ*	−0.1317	0.1549
Consultation and laboratory × respondent works in a faith-based/NGO health facility *μ*	−0.6787	0.3867
Consultation and laboratory × respondent works in a faith-based/NGO health facility *σ*	0.8555	0.6340
Consultation and laboratory × respondent works at secondary care-level facility *μ*	−1.0208^a^	0.2622
Consultation and laboratory × respondent works at secondary care-level facility *σ*	−0.1729	0.2413
Consultation and drugs *μ*	−0.0289	0.2891
Consultation and drugs *σ*	0.4955	0.4415
Consultation and drugs × respondent works in a public health facility *μ*	1.0174^a^	0.3729
Consultation and drugs × respondent works in a public health facility *σ*	0.3589	0.2275
Consultation and drugs × respondent works in a faith-based/NGO health facility *μ*	−0.1133	0.4538
Consultation and drugs × respondent works in a faith-based/NGO health facility *σ*	0.6875	0.5131
Consultation and drugs × respondent works at secondary care-level facility *μ*	−1.0830^a^	0.2911
Consultation and drugs × respondent works at secondary care-level facility *σ*	−0.8934^a^	0.2560
Consultation, laboratory, drugs and imaging *μ*	−1.1372^a^	0.4289
Consultation, laboratory, drugs and imaging *σ*	1.8872^a^	0.2525
Consultation, laboratory, drugs and imaging × respondent works in a public health facility *μ*	1.9920^a^	0.5305
Consultation, laboratory, drugs and imaging × respondent works in a public health facility *σ*	−0.9370^a^	0.4369
Consultation, laboratory, drugs and imaging × respondent works in a faith-based/NGO health facility *μ*	−0.0156	0.6568
Consultation, laboratory, drugs and imaging × respondent works in a faith-based/NGO health facility *σ*	0.1567	0.5381
Consultation, laboratory, drugs and imaging × respondent works at secondary care-level facility *μ*	−1.0329^a^	0.4098
Consultation, laboratory, drugs and imaging × respondent works at secondary care-level facility *σ*	−0.0818	0.3664
Opt-out	0.0600	0.3936
Opt-out × respondent works in a public health facility	−1.2366^a^	0.4918
Opt-out × respondent works in a faith-based/NGO health facility	−0.4219	0.5513
Model fit statistics		
Log-likelihood (final)	−2188.0796	
Observations	8388	
Number of decision-makers (*n*)	233	
Draws (Halton)	1000	

a The 95% confidence interval does not include zero. μ is the mean while σ is the standard deviation. S.E. represents robust standard errors. The coefficients of capitation payment rate per individual per year and its interactions were restricted to a lognormal distribution. The opt-out and its interactions were fixed. All other coefficients and their interactions were normally distributed.

Furthermore, the interaction between the level of care the respondent worked in and all levels of the services to be paid by capitation rate attribute were statistically significant and negative (Table 7). This suggested that, if everything else was held constant, managers who worked at secondary care-level hospitals had a lower preference for a service package that contained a broad range of health services paid through capitation as compared with managers who worked at primary care-level facilities. Conversely, managers who worked at public health facilities had a greater preference for a service package that contained a broad range of health services paid through capitation as compared with managers who worked at private facilities, if everything else was held constant.

Finally, the interaction between the opt-out and ownership of the health facility the respondent worked in revealed interesting results. It suggested that senior health facility managers who worked at public facilities had a significant lower preference for the opt-out (i.e. no capitation) as compared with those at private facilities.

### Latent preference heterogeneity

We unearthed interesting heterogeneity in preferences across three latent classes (Table 8). Managers in Class 1 exhibited preference for the opt-out (i.e. no capitation) and lower preference for capitation arrangements that bundled a broader range of health services compared with those that paid for a narrower set of services (consultations only). In Class 2, managers exhibited lower preference for the opt-out and preference for capitation arrangements that bundled a broader range of services compared with a narrower set of services. In Class 3, managers exhibited lower preference for the opt-out and capitation arrangements that bundled a broader range of health services compared with those that paid for a narrower set of services. The class assignment probabilities are a function of the following covariates: ownership of the health facility the respondent worked in, gender, the level of care the respondent worked in and respondent’s job title (Table 8).

**Table 8:** Latent class model preference estimates

Capitation attribute	Class 1		Class 2		Class 3	
	Coefficient	S.E.	Coefficient	S.E.	Coefficient	S.E.
Payment schedule	−0.1123^a^	0.0239	−0.0918^a^	0.0113	−0.0748^a^	0.0138
Timeliness of payment						
Timely	Ref. (0)		Ref. (0)		Ref. (0)	
Delayed by less than 3 months	−0.5695^a^	0.2080	−0.3546^a^	0.0993	−0.1989^a^	0.1345
Delayed by more than 3 months	−1.4535^a^	0.2593	−1.1156^a^	0.1234	−0.6153^a^	0.1494
Capitation rate per individual per year	0.0004^a^	0.0001	0.0002^a^	0.0001	0.0006^a^	0.0001
Services to be paid by the capitation rate						
Consultation only	Ref. (0)		Ref. (0)		Ref. (0)	
Consultation and laboratory tests	−0.6906^a^	0.2489	0.4229^a^	0.1294	−0.7260^a^	0.1821
Consultation and drugs	−0.7738^a^	0.2734	0.6072^a^	0.1450	−0.8777^a^	0.1973
Consultation, laboratory tests, drugs and imaging	−0.6333^a^	0.2662	0.7114^a^	0.1718	−1.9140^a^	0.2922
Opt-out	2.0954^a^	0.3372	−3.2301^a^	0.5397	−1.0837^a^	0.2294
Prior class membership probability	0.1990		0.4500		0.3510	
Class membership model parameters						
Respondent is head of the facility	−0.4727	0.5265	−1.0027^a^	0.5082	Ref. (0)	
Respondent is the administrator of the facility	−0.3114	0.5260	−0.7142	0.5124		
Respondent works in a private health facility	0.2463	0.5005	−1.8279^a^	0.5371		
Respondent works in a faith-based health facility	−0.0374	0.5481	−2.2800^a^	0.6584		
Respondent works in a secondary care-level health facility	0.3390	0.4819	−1.5263^a^	0.5216		
Respondent is female	0.2223	0.4304	−0.3738	0.4357		
Constant	−0.6270	0.7381	2.7500^a^	0.7308		
Model fit statistics						
Log-likelihood (final)	−2171.0991					
Akaike information criterion	4418.200					
Bayesian information criterion	4549.340					
Observations	8388					
Number of decision-makers (*n*)	233					

a The 95% confidence interval does not include zero. S.E. represents robust standard errors. All coefficients are fixed.

## Discussion

The study set out to unearth the preferences of senior health facility managers in Kenya for capitation payments, the relative importance the respondents placed on the attributes, marginal WTA estimates and heterogeneity in preferences. We found that capitation arrangements with frequent payment schedules, timelier disbursements, higher payment rates per individual per year and paid for a narrow set of healthcare services were preferred. The capitation rate per individual per year was the most important attribute followed by payment schedule. Furthermore, senior health facility managers were willing to accept an increase in the capitation rate to compensate for payments delayed by more than 3 months as compared with those that were paid on time. Moreover, they were willing to accept an increase in the rate to compensate for infrequent payment schedules and bundling a broader set of health services as compared with a narrow set. Finally, the results suggested the presence of inter-respondent heterogeneity in preferences and marginal WTA estimates.

Higher capitation rates per individual per year were preferred by senior health facility managers. Furthermore, this attribute was the most important according to them. These findings reinforce the importance of the capitation rate paid to healthcare providers by purchasing organizations. Robyn *et al.* (2012) found similar results in Burkina Faso where higher capitation payment levels were preferred by health workers. A higher capitation rate means more revenue for health facilities. Therefore, it would enable them to not only invest in providing services to patients but also pay salaries and make infrastructural improvements at the health facility (Obadha *et al.*, 2019c). Preference for a higher capitation rate was expected as Kenyan healthcare providers have voiced their concerns over inadequacy of the rates to cover the cost of services provided to NHIF enrolees. The NHIF currently pays health facilities KES 1200 (US $1.2) for an enrolee a year for those covered under the national scheme. Sieverding *et al.* (2018) found that Kenyan private providers perceived NHIF capitation rates per enrolee as inadequate. In Nigeria, Etiaba *et al.* (2018) also note that capitation rate from the Formal Sector Social Health Insurance Program of the National Health Insurance Scheme was deemed inadequate by public and private providers. However, it has been reported that the perception by healthcare providers in Kenya that capitation rates are low may be because of their limited understanding of how a capitation payment mechanism works (Obadha *et al.*, 2019c). There is the misunderstanding that a capitation payment rate is expected to cover the cost of individual care seeking events rather than it being a rate that cumulatively covers the risk of the capitated population (Obadha *et al.*, 2019c). It has also been reported that healthcare providers in Kenya do not have access to the list of enrolees registered at their health facilities, making capitation payments unpredictable and heightening perceptions of the inadequacy of capitation payment rates (Obadha *et al.*, 2019c).

Senior health managers preferred capitation payments that bundled a narrow range of health services (leaving the other services to be paid for separately using another payment mechanism such as FFS or case-based mechanism) compared with those that bundled a broader range of services under the singular payment (capitation). Capitation in general creates incentives for healthcare providers to underprovide services. Therefore, bundling a wide range of health services under a capitation payment arrangement might not be desirable for providers. This explains why senior health facility managers in our study were willing to accept an increase in the capitation rate to compensate for bundling a broader set of health services in the capitation payment. These results are similar to those of a laboratory experiment by Hennig-Schmidt *et al.* (2011) who found that physicians provided a smaller quantity of health services under capitation than FFS. Furthermore, a field experiment by Brosig-Koch *et al.* (2016) in Germany confirmed these findings. In Kenya, a study by Wangai *et al.* (2019) found that healthcare providers had to compromise on the quality of services provided under capitation when the number of visits from enrolees increased.

Moreover, we found that managers who worked at public health facilities as compared with those working at private facilities had a lower preference for higher capitation payment rates and a preference for capitation arrangements that bundled a broader range of healthcare services (consultation, laboratory tests, drugs and imaging) compared with those that paid for a narrower range of services (consultations only). It did not mean that public providers wanted to be paid less, rather private providers wanted to be paid more. Private and faith-based facility managers must procure drugs, medical equipment and pay salaries and therefore factor these when calculating the cost of health services. To them, providing a wide range of health services under capitation, which pays a fixed rate per individual, might lead to losses. Suchman (2018) found that private providers in Kenya limited the number of health services provided to NHIF enrolees under capitation. Conversely, in addition to capitation payments from the NHIF, public providers do receive line-item budgets, medical supplies, drugs, equipment and staff salaries from county governments. This might explain why public health facility managers manifested altruistic behaviour as they prioritized service provision ahead of profits.

In addition, managers who worked at secondary care-level hospitals had a lower utility for capitation arrangements that bundled a broader range of services as compared with those who worked at primary care-level facilities. This might be explained by the fact that secondary care-level hospitals provide highly specialized and broader range of services than primary care-level facilities and are more sensitive to the costs of providing these services. Therefore, bundling a broader range of services such as highly specialized laboratory tests and drugs to be paid using a fixed capitation rate might not be appealing to them from a cost perspective. Therefore, they would have preferred services to be unbundled and paid individually using other PPMs such as FFS. Moreover, secondary care-level health facilities including public facilities, charge user fees for those patients who do not have health insurance.

Frequent capitation disbursements were preferred in our study. Senior health facility managers did not want to wait long to receive the payments in the case they depleted their current funds. These results are consistent with the findings of Robyn *et al.* (2012) in Burkina Faso who established that capitation payments disbursed four times and twice a year were preferred to those disbursed only once. Moreover, timely capitation payments were strongly preferred by senior health facility managers in our study. Timely disbursements mean that salaries can be paid on time and drugs and medical commodities restocked, which may create positive incentives to improve health service delivery (Obadha *et al.*, 2019c). Delayed payments affect health facility operations and may lead to providers either turning away enrolees or charging them informal fees. This has been reported in different settings such as Ghana and Nigeria (Dalinjong and Laar, 2012; Etiaba *et al.*, 2018; Suchman, 2018).

Overall, there was a lower preference for the opt-out. However, public health facility managers seemed to dislike the opt-out more than those at private and faith-based facilities suggesting that they might have liked capitation. Private and faith-based health facilities may have contracts with different insurance companies and get paid through different mechanisms such as FFS and case-based payments. They are therefore more ‘picky’ when it comes to what is appropriate in capitation. Public health facilities on the other hand might only have NHIF as the only insurance company they have a contract with. Therefore, they might have preferred some form of capitation to nothing at all as it represented a revenue source.

Our findings have several policy implications. First, to address the perception that the capitation rate offered by the NHIF is low, there is a need to review the current capitation rates, informed by costing evidence, to ensure that they are adequate. This is especially because it has been observed that the process of provider payment rate development by the NHIF is not informed by robust evidence (Munge *et al.*, 2018). However, reviewing the capitation rate alone may not be enough to shift healthcare provider perceptions that the rate is inadequate. The NHIF will also need to reform its PPM development process to ensure that healthcare providers are adequately engaged and informed to enhance their understanding of the mechanism and their buy-in. Second, it has been reported that capitation payments by the NHIF to health facilities are often delayed (Obadha *et al.*, 2019c). Improving the frequency and timelines of capitation disbursements would go a long way in motivating healthcare providers to deliver needed services and improve quality and efficiency. Third, the NHIF could consider reviewing its current capitation mechanism to determine whether there is scope to unbundle certain services to be paid for separately. This will disincentivize skimping of care that may arise when providers have the perception that the range of bundled services under the capitation payment is not financially viable. Fourth, given that trade-offs will have to be made over the relative extent to which the attributes of the capitation mechanism are reformed, the NHIF should complement these with strengthening monitoring mechanisms to guard against undesired provider behaviour incentivized current and future capitation arrangements.

This study has several strengths. First, we collected preference data from senior health facility managers spread across seven counties that were randomly selected and therefore a good representation of the Kenyan context. Our findings can also be applicable to similar contexts. Second, we collected preference data from senior managers who worked at health facilities that were NHIF accredited and those not accredited, which enriched our results. Moreover, the DCE methodology ensured that we could study capitation alternatives that did not yet exist.

However, the study had some limitations. Since it was a DCE presenting hypothetical choice sets to respondents, it might have been prone to hypothetical bias. Second, capitation is mainly used by the NHIF. Therefore, study participants’ responses focused on NHIF’s capitation arrangement rather than the payment mechanism in general even if it was used by another purchaser. Third, there are some unobserved factors that may have influenced our results. For instance, there is anecdotal evidence of informal payments in the Kenyan health system, which may or may not have influenced provider preference for capitation payment rates. Nevertheless, the study provided useful pointers for purchasers to think about when reforming capitation payment mechanisms.

## Conclusions

In conclusion, as Kenya reforms its health financing policies by modifying its PPMs, then it is important to focus on senior health facility managers’ preferences for capitation attributes. These attributes can be used as potential targets for interventions aimed at configuring capitation and reorienting the health system towards achieving UHC by 2022. There may be need to review capitation rates, improve the timeliness of disbursements and increase the frequency of payments to create positive incentives for healthcare providers to deliver needed services and improve quality and efficiency.
